# Protocatechuic acid as an inhibitor of the JNK/CXCL1/CXCR2 pathway relieves neuropathic pain in chronic constriction injury rats

**DOI:** 10.17305/bjbms.2021.5928

**Published:** 2021-11-23

**Authors:** Hong-xia Chang, Yue-feng Zhao

**Affiliations:** 1 Department of Anesthesiology, Shangluo Central Hospital, Shangluo, Shaanxi Province, China,; 2 Department of Neurosurgery, Shangluo Central Hospital, Shangluo, Shaanxi Province, China

**Keywords:** Chronic neuropathic pain, JNK/CXCL1/chemokine receptor 2 signaling pathway, protocatechuic acid, tumor necrosis factor-α

## Abstract

Emerging evidence has shown that protocatechuic acid (PCA) has antioxidant and anti-inflammatory effects. Evidence suggests that PCA can alleviate the injury of sciatic nerve, while the mechanism of its therapeutic effect on neuralgia remains unknown. Chromium bowel ligation was used *in vivo* to establish a chronic constriction injury (CCI) rat model to induce sciatic nerve pain. Subsequently, two doses of PCA were used to treat CCI rats. *In vitro*, 10 ng/mL tumor necrosis factor-α (TNF-α) was used to stimulate glial satellite cells derived from the dorsal root ganglia (DRG) L4-L6 of the sciatic nerve to simulate sciatic nerve pain. PCA relieved mechanical allodynia and thermal hyperalgesia in CCI rats. CCK-8 assay revealed that PCA inhibited the proliferation of glial satellite cells induced by TNF-α. Moreover, enzyme-linked immunosorbent assay demonstrated that PCA could improve the inflammatory response of rats caused by CCI and cells induced by TNF-α. Next, reverse transcription quantitative polymerase chain reaction and Western blot assays showed that PCA blocked the c-Jun N-terminal kinase/the chemokine ligand 1/CXC chemokine receptor 2 (JNK/CXCL1/CXCR2) pathway by inhibiting CXCL1 levels in cells induced by TNF-α and DRG in CCI rats. In conclusion, PCA can alleviate neuropathic pain in CCI rats and improve oxidative stress by inhibiting the JNK/CXCL1/CXCR2 signaling pathway. Thus, these findings provide a new perspective for the treatment of neuropathic pain caused by CCI.

## INTRODUCTION

Neuropathic pain is caused by the damage or dysfunction of the somatosensory nervous system, usually accompanied by allodynia and hyperalgesia [1]. Although surgical treatment and conservative drug treatment are widely used, the effect is not satisfactory. In recent years, more and more researchers advocate the use of natural drugs or extracts to stimulate the repair and proliferation of injured nerves. It was reported that combined immunosuppressive therapy with fibrin catheter and mesenchymal stem cells promoted regeneration after peripheral nerve injury [2]. Clinically, losartan alleviated neuroinflammation and neuropathic pain after spinal nerve ligation [3]. Brazilein inhibited the activation of PSD-95 in the corresponding segment of the sciatic spinal cord after sciatic nerve injury in BALB/c mice, thereby inhibiting the overexpression of free radicals and promoting the regeneration of myelin sheath [4]. In addition, acetyl-11-keto-β-Boswellic acid regulated the proliferation of Schwann cells and the formation of myelin sheath by upregulating the phosphorylation of ERK to promote the repair of sciatic nerve injury in rats [5]. Although clinical drugs have some advantages in the treatment of neuropathic pain, there is no doubt that they have side effects. A variety of plant extracts and monomers exert anti-neuropathic pain effects through anti-inflammatory and anti-oxidant effects. Moreover, simvastatin inhibited neuronal apoptosis after spinal cord injury by activating the Wnt/β-catenin signaling pathway, and relieved sciatic nerve crush neuropathic pain in rats [6].

Protocatechuic acid (PCA) is one of the main benzoic acid derivatives in vegetables and fruits. It also exists in tea, hibiscus flower, *Salvia miltiorrhiza*, and other Chinese herbal medicines. It has the functions of anti-oxidation, anti-bacterial and anti-hyperglycemia [7-9]. In addition, it promoted the proliferation of neural stem cells and reduced the apoptosis of basal cells [10], and the progression of atherosclerosis was inhibited by PCA treatment via upregulation of MER proto-oncogene tyrosine kinase in mice [11]. PCA inhibited the proliferation of airway smooth muscle cells and extracellular matrix (ECM) protein deposition mediated by transforming growth factor-α by inhibiting the small mothers against decapentaplegic2/3 (Smad2/3) signal pathway, thus inhibiting airway remodeling in asthma [12]. It also inhibited the production of ­inflammatory mediators in keratinocytes stimulated by lipopolysaccharide by reducing the toll like receptor 4-dependent activation of the Akt, mTOR, JNK, and p38 MAPK [9]. PCA can significantly reduce serum malondialdehyde (MDA), tumor necrosis factor (TNF)-α, and kidney MDA levels in renal ischemia-reperfusion rats, and increase serum and kidney TAS and superoxide dismutase (SOD) levels [13]. Moreover, it can reduce the ratio of degeneration, oxidative damage, GSH depletion, and microglia activation of neurons in hippocampus and cortex [14]. This study aims to investigate the mechanism of PCA’s alleviation of sciatic nerve injury pain. Emerging evidence confirms that chemokines, including chemokine (C-X-C motif) ligand 1 (CXCL1), enhance cell proliferation and invasion in high-grade prostate cancer, gastric cancer, and other cancers [15,16]. CXCL1 protein level was increased in the model of neuropathic pain induced by L5Tx in BALB/c mice [17]. One study reported that the CXCL1/CXC chemokine receptor 2 (CXCR2) axis plays an important role in treatment of nerve pain [18,19]. In addition, a study reported that blocking CXCL1 and its receptor CXCR2 by the spinal cord inhibited paclitaxel-induced peripheral neuropathy in mice [20]. The previous studies have verified that CXCL1 chemokine participates in neuropathic pain by activating CXCR2 in spinal microglia [18,21]. CXCR2 has been reported as a target for the treatment of chronic demyelinating diseases such as multiple sclerosis [22]. The purpose of this study was to investigate whether PCA can alleviate sciatic nerve pain by intervening the CXCL1/CXCR2 axis.

## MATERIALS AND METHODS

### Animals

Male SD rats (weight 180–200 g) provided by the experimental animal center of Shangluo central hospital. Rats were placed in separate cages (humidity 60 ± 5%; temperature 23 ± 4°C, light/dark cycle 12–12 h).

### Chronic Constriction Injury (CCI) Rat Model

The establishment of CCI induced neuropathic pain rat model was based on previous reports [23,24]. In brief, after preoperative measurement of exercise behavior, 1% pentobarbital sodium was injected into the peritoneum of rats. In the aseptic condition, the biceps femoris and the sciatic nerve stem were directly passivated by 4-0 chromium gut ligation (about 1 mm away) to dissect the biceps femoris [25]. Rats in the sham group underwent the same operation without ligation. After suturing, ligation of the sciatic nerve was observed, the diameter of the tubercle was slightly reduced, and there was twitch on the right hind limb. The ligation should not be too tight to prevent the nerve ligation completely blocking the surrounding blood membrane. Pain threshold reduction and walking disorder were used to confirm the success of the model. Then the incision was sutured, and all the animals returned to the cage to drink and eat freely. Pain threshold reduction and walking disorder were used to confirm the success of the model. Two days (48 h) after the surgery, the rats were euthanized and the left dorsal root ganglion (DRG) L4-6 was removed for follow-up study.

### Animal Grouping and Treatment

Forty-eight male SD rats (6–8 weeks old, weighing 180–200 g) were randomly divided into six groups, including blank control (ctrl) group; sham group; CCI group. The rats in one CCI group were orally administrated with 30 mg/kg/d simvastatin (TOCRIS, Bristol, UK), while rats in the other CCI group were orally administrated with 20 or 50 mg/kg/d PCA (≥97% pure, Sigma-Aldrich, Corp., St. Louis, MO, USA) (n = 8 each group). In the sham group, sciatic nerve was only isolated and no injury was found. Simvastatin was used as positive control, PCA as treatment group, and equal volume of normal saline as blank control for 21 days. Stock solution of PCA and simvastatin was freshly prepared every other day using normal saline as a vehicle.

### Thermal Sensitivity Test

Thermal withdrawal latency (TWL) was used to measure the latency of response to thermal stimulation of the hind paw [26]. This test was measured 1 day before surgery and days 7 and 14 after surgery. Rats were placed in transparent Plexiglass cages where the foot lifting reflex was observed when the lower limbs of rats were irradiated with the plantar beam, and the time of foot lifting reflex was recorded. The cut off time was 25 s. To prevent foot skin injury, stimulation was performed every 15 min and repeated 3 times.

### Mechanical Nociceptive Test

Paw withdrawal mechanical threshold (PWMT) was measured by von Frey filament method day 1 before surgery, days 7 and 14 after surgery. In brief, dynamic plantar anesthesia (Ugo-Basile) was used to measure mechanical hyperalgesia. Rats were placed on a wire mesh platform in a separate Plexiglass compartment (8.5 cm long and 3.4 cm high) and allowed to adapt for about 1 h. Subsequently, the mechanical stimulus was transferred from under the floor of the test room to the plantar surface of the hind paw of the rat through an automatic testing device. An electronic lifting force (0–5 g, 35 s) was used to push the steel bar (2 mm). A sudden withdrawal of the paw or licking in response to von Frey filament stimulation was considered a positive response. If a positive response was observed, a filament with a lower force was applied, and if no response occurred, a filament with a higher force was used. The threshold values were the maximum pressure (in grams) applied to trigger the retraction of the paw. Each rat was measured 3 times with an interval of 3–5 min, and the average value was taken as the final PWMT [27]. The mechanical stimulation would automatically withdraw, when the animal retracted its hind paw. The nociceptive response of mechanical sensitivity was mechanical paw lifting.

### Cell Culture and Drug Intervention

To anesthetize the rats, 4% sodium pentobarbital solution (60 mg/kg) was injected intraperitoneally. At the end of the heat sensitivity test (on the 21^st^ day of drug treatments), rats were anesthetized by intraperitoneal injection of 4% pentobarbital sodium solution at a dose of 60 mg/kg, then euthanized. The extraction and culture of primary glial satellite cells from L4 to L6 spinal cord segments of rats were described previously [28,29]. First, the spine of each group of rats was taken, the spinal cord muscle was removed, and then both sides of the spinous process were cut open with ophthalmic scissors. Under the stereo microscope, the blood vessels and nerve fibers in the spinal cord cavity were completely removed, and then the DRG was clamped under the stereo microscope, and the remaining nerve fibers and capsules were gently removed with the fiber clamp. DMEM/F12 (1:1) (Invitrogen, Carlsbad, CA, USA) containing penicillin/streptomycin was used to collect the treated DRG in 35 mm Petri dish on ice. A sufficient number of DRGs (about 54) were collected in 35 mm plates, then inoculated into 6-well plates containing satellite glial cell medium (500 μL/well) and cultured in a humidified 5% CO_2_ incubator at 37°C. After 3 days of culture, some cells migrated from DRG. After subculture, the expression of specific marker glial fibrillary acidic protein (GFAP) was detected. After inducing satellite glial cell activation, drug intervention was given.

In the TNF-α stimulation experiment, the complete medium was replaced by the Opti-MEM medium. Then TNF-α (10 ng/mL) or the corresponding solvent was added into the Opti-MEM medium. Then, the cells were collected after 1 h of stimulation.

In the intervention experiment of the MAPK signal pathway, first, the complete medium was replaced with Opti-MEM medium, and then PCA (20 or 50 μM), the JNK pathway inhibitor SP600125 (20 or 50 μM), the MEK inhibitor U0126 (20 or 50 μM) or the p38/MAPK pathway inhibitor SB203580 (20 or 50 μM) was added it. They were added 30 min in advance, subsequently glial satellite cells were stimulated with TNF-α or solvent for 1 h, and these cells were cells.

### Enzyme-linked Immunosorbent Assay (ELISA)

The protein lysate was added to each group of spinal cord tissue and satellite glial cell samples to homogenize tissues and cells. The supernatant was collected after centrifugation at ×8,000 g at 4°C. The contents of SOD, MDA, interleukin (IL)-1β, IL-6, and TNF-α in rat DRG and glial satellite cells were determined by ELISA kits (Thermo Fisher Scientific, Waltham, MA, USA), according to the manufacturer’s instructions. A microplate reader (Awareness Technology, Inc, Palm City, FL, USA) was used to measure the absorbance of the sample at 450 nm and calculate the protein concentration based on the standard curve.

### Reverse Transcription Quantitative Polymerase Chain Reaction (PCR)

Total RNA was extracted from cells and tissues using TRIzol reagent (Invitrogen, Carlsbad, CA, USA) according to the manufacturer’s protocol. Reverse transcription was performed with a Script Reverse Transcription kit (Qiagen, Dusseldorf, Germany). Real-time PCR analysis was conducted with the SYBR Premix Ex Taq II (Takara, Dalian, China) on an ABI 7500 Real-Time PCR System (Applied Biosystems, Waltham, MA, USA) under the following conditions: 95°C for 1 min followed by 35 cycles of 95°C for 20 s, then 56°C for 10 s, and 72°C for 15 s. The sequences of primers were as follows: CXCL1 forward, 5’-GGC TGG GAT TCA CCT CAA GAA-3’; and reverse, 5’-TGT GGC TAT GAC TTC GGT TTG-3’; CXCR2 forward, 5’-TTC TGA CCC GCC CTT TAC TCT GT-3’; and reverse, 5’-CGC AGT GTG AAC CCA TAG CAG-3’; GAPDH forward, 5’-TGC AGT GGC AAA GTG GAG ATT-3’; and reverse, 5’-TCG CTC CTG GAA GAT GGT GAT-3’. Finally, the relative expression levels of CXCL1 and CXCR2 were calculated with the 2^-DDCt^ method.

### Western Blotting

RIPA lysis buffer (CW Biotech, Beijing, China) was used to lyse the cells and tissues, and the total protein was extracted. The protein concentration was determined by the BCA protein assay kit (Thermo Fisher Scientific, Waltham, MA, USA). About 10% SDS-PAGE was used to separate protein samples in equal amounts. Next, the complex was transferred and sealed onto a nitrocellulose membrane (General Electric Co, USA). After being blocking with 5% skim milk for 2 h at room temperature, the membranes were incubated overnight at 4^o^C with the following primary antibodies: Rabbit polyclonal to JNK antibody (ab112501, 1:300) (Abcam, Cambridge, UK), rabbit polyclonal to JNK1+JNK2 (phospho T183 + Y185) (ab131499, 1:300, abcam), rabbit polyclonal to CXCL1 (ab86436, 1:500, abcam), rabbit polyclonal to CXCR2 (ab14935, 1: 300, abcam), rabbit polyclonal to ERK1 + ERK2 (ab17942, 1: 300, abcam), rabbit polyclonal to ERK1 (phospho Y204) + ERK2 (phospho Y187) (ab47339, 1: 300, abcam), rabbit anti-CREB antibody (ab31387, 1:400, abcam), and rabbit anti-c-Fos antibody (ab190289, 1:300, abcam). After washing 3 times for 10 min each with TBST, the membranes were incubated with horseradish peroxidase-conjugated goat anti-rabbit IgG H and L (HRP) (ab205718, 1: 2,000, abcam) for 1 h. Signals were visualized by an enhanced chemiluminescence detection reagents (Habersham, Little Chalfont, United Kingdom) and analyzed with ImageJ software (National Institutes of Health, Bethesda, MD, USA). GAPDH was used as an endogenous control to normalize protein expression. The fold change versus control group was the demonstration of target proteins’ relative expression levels.

### Cell Viability Assay

First, 100 mL of cell suspension was placed on 96 well plate and cultured in 37°C, 5% CO_2_ incubator for 24 h. Subsequently, 10 mL of the test substance was added to the culture plate, and the culture plate was incubated in the incubator for 24, 48, and 72 h, respectively. Afterward, 10 μL of CCK-8 solution (Dojindo Laboratories, Japan) was added to each hole, and then the culture plate was incubated in the incubator for 1 h. The absorbance at 450 nm was measured using a microplate reader (Molecular Devices, Shanghai, China).

### Ethical Statement

These animal surgeries were carried out in accordance with the guidelines of the National Institutes of health on animal care. This study was approved by the Department of Shangluo central hospital on April 5, 2018.

### Statistical Analysis

SPSS 19 software (IBM-SPSS, Armonk, NY, USA) was used for statistical analysis. All data were expressed. Student’s t-test was performed to compare the differences between two groups, analysis of variance (ANOVA) was performed for the comparison among groups, and multiple comparisons were performed using Tukey-Kramer correction. p<0.05 was considered to be statistically significant.

## RESULTS

### PCA Alleviated Mechanical Allodynia and Thermal Hyperalgesia in CCI Rats

**FIGURE 1:**
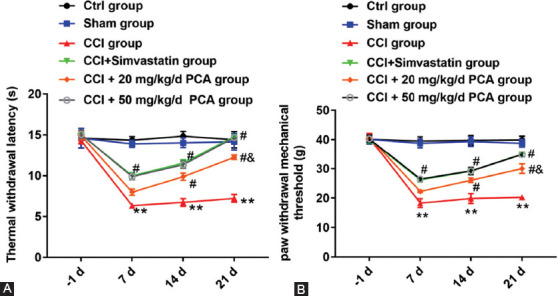
Effect of protocatechuic acid (PCA) on the thermal hypersensitivity and mechanical hyperalgesia in chronic constriction injury (CCI) rats. Chromium bowel ligation was used to establish a CCI rat model to induce sciatic nerve pain. SD rats were divided into six groups, including ctrl group, sham group, CCI group and CCI rats administrated with simvastatin or two doses (20 or 50 mg/kg) of PCA group. TWL (A) and PWMT (B) indicators were used to confirm the success of the model. We detected the TWL and PWMT in six groups of SD rats (eight in each group). Data were presented as mean ± SEM of three independent experiments. **p<0.01 versus ctrl group or sham group, ^#^p<0.05 versus CCI group, ^&^p<0.05 versus CCI group treated with simvastatin or 50 mg/kg PCA.

To explore the effect of PCA on the pain threshold of rats, we measured and recorded the response of rats in each group to mechanical and thermal stimulation from the day before surgery to day 21 after surgery (days −1, 7, 14 and 21). As shown in Figure 1A and B, the TWL value of rats in the CCI group was lower than those in the sham group or the control group, and mechanical allodynia was observed in CCI rats on days 7, 14, and 21 (p<0.01). However, compared with the CCI group, PCA treatments significantly increased TWL and PWMT values, and 50 mg/kg PCA was better than 20 mg/kg PCA (TWL: F5,18 (d7) = 204.097; F5,18 (d14) = 103.330, F5,18 (d14) =62.884; p<0.05) (PWMT: F5, 18 (d7) = 180.456; F5, 18 (d14) = 91.305, F5, 18 (d14) = 125.226; p< 0.05). The results showed that PCA improved TWL and mechanical allodynia in rats caused by CCI.

### PCA Alleviated the Inflammation in CCI Rats

**FIGURE 2:**
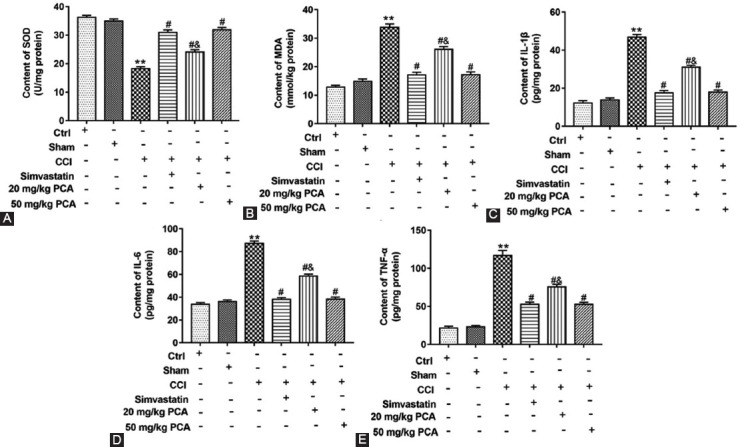
Effects of protocatechuic acid (PCA) on oxidative stress and inflammation of L4-6 spinal cord segments in chronic constriction injury (CCI) rats. SD rats were treated the same as thermal sensitivity test. The contents of SOD (A), MDA (B), IL-1β (C), IL-6 (D) and tumor necrosis factor-α (E) in rat tissues were detected by ELISA. **p<0.01 versus ctrl group or sham group, ^#^p<0.05 versus CCI group, ^&^p<0.05 versus CCI group treated with simvastatin or 50 mg/kg PCA.

Then, we explored the effects of different treatments on the oxidative stress and inflammatory mediators of L4-6 DRG. As shown in Figure 2A, CCI rats decreased the content of SOD compared with ctrl group or sham group (p<0.01), while PCA treatment increased (F_5,12_=55.94; p<0.05). However, the contents of MDA, IL-1β, IL-6, and TNF-α were increased in CCI rats compared with ctrl group or sham group (F_5, 12(MDA)_=212.60; F_5, 12(IL-1_^b^_)_ = 399.80; F_5, 12(IL-6)_=196.80; p<0.01), while PCA treatment reduced these indexes (p<0.05), and the therapeutic effect of 50 mg/kg PCA was better than that of 20 mg/kg PCA (p<0.05) (Figure 2B-E). These results suggested that the immune function in the nerve tissue of rats with different treatments had changed, and PCA could restore the inflammation caused by CCI treatment.

### PCA Inhibited the Proliferation of Rat DRG Glial Satellite Cells Treated with TNF-α

**FIGURE 3:**
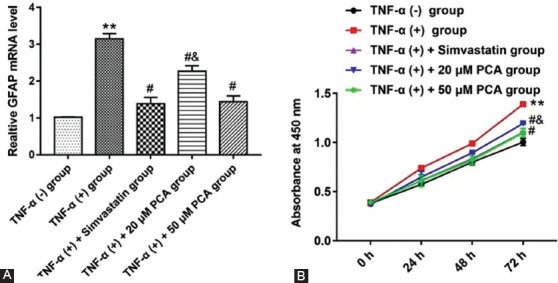
Effects of protocatechuic acid (PCA) in different doses on the activity and behavior of glial satellite cells induced with tumor necrosis factor (TNF)-α derived from L4-6 spinal cord segments. Cells induced by TNF-α were treated with simvastatin or PCA (20 or 50 μM), respectively. (A) Reverse transcription quantitative polymerase chain reaction was used to detect the activity marker GFAP level in glial satellite cells. (B) Cell proliferation was detected with CCK-8 assay. **p<0.01 versus TNF-α (-) group, ^#^p<0.05 versus TNF-α (+) group, ^&^p< 0.05 versus TNF-α (+) group treated with simvastatin or 50 μM PCA.

We used TNF-α to stimulate glial satellite cells derived from DRG of rats to explore the effect of different treatments on cell behavior. Glial satellite cells induced by TNF-α were treated with simvastatin, 20 or 50 μM PCA. We detected the GFAP mRNA level in glial satellite cells treated with five different treatments (Figure 3A) and found that the mRNA level of GFAP in TNF-α treated glial satellite cells was increased, while PCA could reverse the promoting role (F4,25=17.60; p<0.01). GFAP was an important active market of glial satellite cells, so we could speculate that TNF-α could activate glial satellite cells. As shown in Figure 3B, TNF-α stimulated the proliferation of glial satellite cells compared with the untreated group (p<0.01), while two doses of PCA reversed astrocyte proliferation (F_4,10(24 h)_=14.57; F_4,25(48 h)_= 21.94; F_4,25(72 h)_ = 131.2; 67.38; p<0.05), and the effect of 50 μM PCA was better than that of 20 μM PCA. These results indicated that PCA could inhibit the promotion of TNF-α on cell proliferation.

### PCA Alleviated the Inflammation in Glial Satellite Cells Induced by TNF-α

**FIGURE 4:**
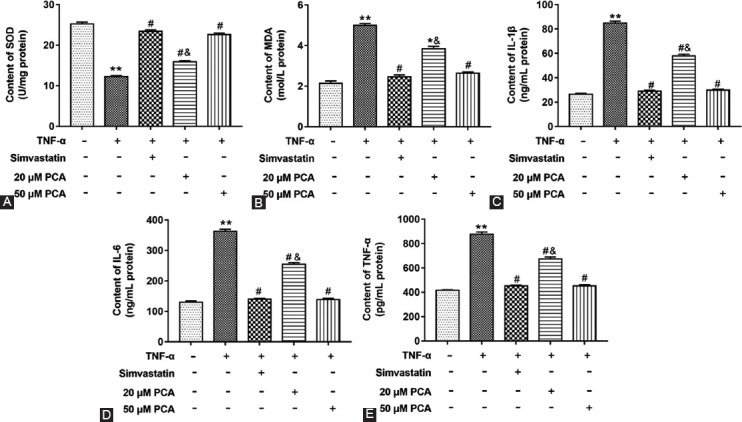
Effects of protocatechuic acid (PCA) on oxidative stress and inflammation in tumor necrosis factor (TNF)-α treated glial satellite cells. Cells were treated the same as cell proliferation assay. The contents of SOD (A), MDA (B), IL-1β (C), IL-6 (D) and TNF-α (E) in cells were detected by using ELISA kits. **p<0.01 versus TNF-α (-) group, ^#^p<0.05 versus TNF-α (+) group, ^&^p< 0.05 versus TNF-α (+) group treated with simvastatin or 50 μM PCA.

Furthermore, we verified whether the trend of inflammatory factors at cell level is consistent with that of DRG. Glial satellite cells were treated with TNF-α alone, TNF-α together with simvastatin and TNF-α together with 20 or 50 μM PCA. We found that the contents of MDA, IL-1β, TNF-α, and IL-6 were increased, and SOD content was decreased in TNF-α treated glial satellite cells compared with untreated group (F_4,10(MDA)_=33.20; F_4,10(IL-1β)_=321.20; F_4,10(TNF-α)_=110.20; F_4,10(IL-6)_=170.60; F_4,10(SOD)_=49.96; p<0.01) (Figure 4A-E). Similarly, two doses of PCA could restore the effect of TNF-α stimulation compared with the TNF-α treated group (p<0.05). These results confirmed that PCA could alleviate the inflammation induced by TNF-α stimulation.

### PCA Downregulated the Expression of CXCL1 mRNA and Protein in Glial Satellite Cells Induced by TNF-α

**FIGURE 5:**
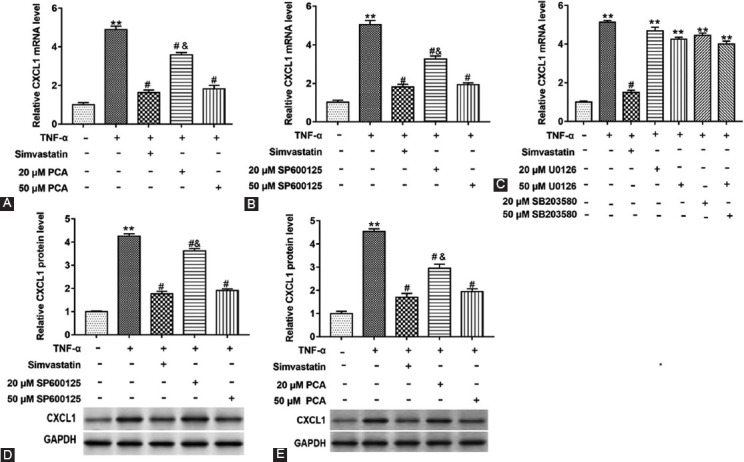
Effect of protocatechuic acid (PCA) on the CXCL1 mRNA and protein level in glial satellite cells treated by tumor necrosis factor (TNF)-α. Glial satellite cells induced by TNF-α were treated with simvastatin, PCA (20 or 50 μM), SP600125 (a inhibitor of the JNK pathway), U0126 (a inhibitor of the ERK pathway) or SB203580 (a inhibitors of the p38/MAPK pathway). Reverse transcription quantitative polymerase chain reaction and Western blot assays were used to detect the CXCL1 mRNA level (A-C) or protein level (D and E) in glial satellite cells. **p<0.01 versus TNF-α (-) group, ^#^p<0.05 versus TNF-α (+) group, ^&^p<0.05 versus TNF-α (+) group treated with simvastatin, 50 μM PCA or SP600125.

Next, we examined CXCL1 mRNA and protein levels, a pain related chemokine, and explored the mechanism of PCA on TNF-α treated glial satellite cells. SP600125, U0126, and SB203580 are inhibitors of the JNK, the ERK, and the p38 MAPK signaling, respectively. Then, glial satellite cells induced by TNF-α were treated with simvastatin, PCA (20 or 50 μM), or SP600125 (20 or 50 μM). CXCL1 mRNA level (Figure 5A) was upregulated in TNF-α treated glial satellite cells (p<0.01), while two doses of SP600125 or PCA downregulated CXCL1 mRNA level (Figure 5A and B) (F4,10=321.60; 376.50; p<0.01). Subsequently, U0126 or SB203580 was used instead of SP600125, while it did not affect the CXCL1 mRNA level (Figure 5C) (p>0.05), which suggested that PCA might block signal transduction in the JNK pathway. Furthermore, we found that CXCL1 protein level was upregulated in cells induced by TNF-α (Figure 5D) (p<0.01). 20 or 50 μM PCA significantly downregulated CXCL1 protein level in cells induced by TNF-α, which was similar to 20 μM SP600125 or 50 μM SP600125 (Figure 5D and E) (F4,10=1261.00; 722.50; p<0.05). In addition, PCA or SP600125 reversed the promoting effect of TNF-α on CXCL1 mRNA and protein levels. It could be concluded that TNF-α could increase CXCL1 mRNA and protein level by activating the JNK signaling pathway, and PCA could block the JNK signal pathway.

### PCA Downregulated CXCR2 mRNA and Protein Levels in Glial Satellite Cells Induced by TNF-α

**FIGURE 6:**
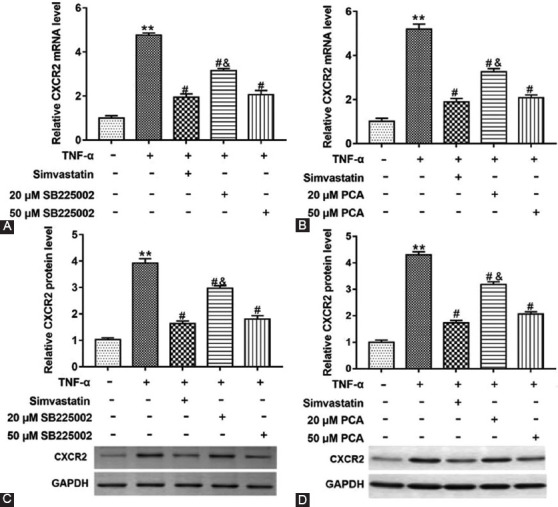
Effect of protocatechuic acid (PCA) on CXCR2 mRNA and protein level in tumor necrosis factor (TNF)-α treated glial satellite cells. Cells induced with TNF-α were treated with simvastatin, SB225002 (20 or 50 μM) or PCA (20 or 50 μM). Reverse transcription quantitative polymerase chain reaction and Western blot assays were used to detect CXCR2 mRNA (A and B) and protein levels (C and D) in glial satellite cells. **p<0.01 versus TNF-α (-) group, ^#^p<0.05 versus TNF-α (+) group, ^&^p<0.05 versus TNF-α (+) group treated with simvastatin or 50 μM PCA or SB225002.

To further explore the mechanism of CXCL1, we studied the mRNA and protein levels of CXCL1 receptor CXCR2 in different glial satellite cells. Cells induced by TNF-α were treated with simvastatin, PCA (20 or 50 μM) or SB225002 (20 or 50 μM) (an antagonist of CXCR2). CXCR2 mRNA and protein levels were also upregulated in cells induced by TNF-α, and 20 or 50 μM SB225002 or PCA downregulated CXCR2 mRNA and protein levels. (F_(4,10) mRNA_=351.40; 287.10; F_(4,10) protein_=292.60; 553.50; P < 0.05) (Figure 6A-D). Therefore, we can speculate that PCA plays a role in CXCR2 by inhibiting CXCL1 in cells.

### PCA Downregulated the JNK/CXCL1/CXCR2 Pathway Related Protein Levels in TNF-α Induced Glial Satellite Cells

**FIGURE 7:**
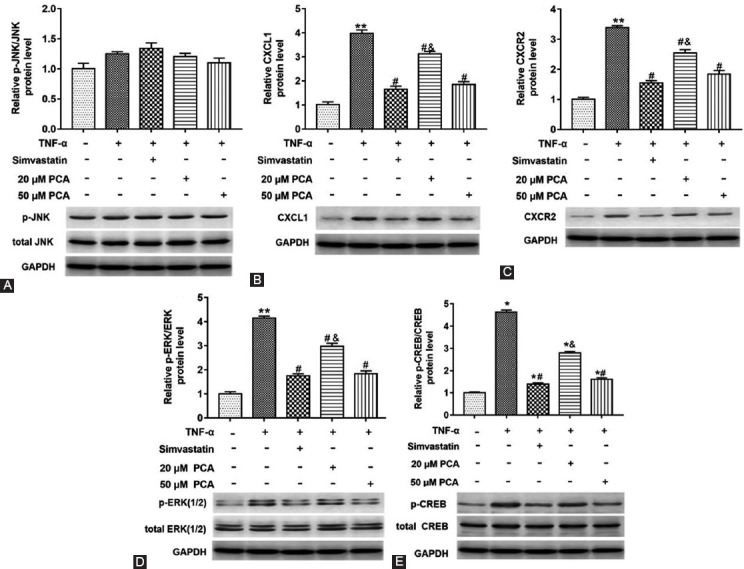
Effect of protocatechuic acid (PCA) on the JNK signaling pathway related proteins in tumor necrosis factor (TNF)-α induced glial satellite cells. Cells induced with TNF-α were treated with simvastatin, or PCA (20 or 50 μM). Western blot assay was used to measure the phosphorylation of ERK, CREB and c-fox protein levels in cells (A-E). **p<0.01 versus TNF-α (-) group, ^#^p<0.05 versus TNF-α (+), ^&^p<0.05 versus TNF-α (+) group treated with simvastatin or 50 μM PCA.

To further explore the effect of PCA on the protein expression of the JNK signaling pathway, we explored the effect of PCA on the protein expression in the upstream and downstream pathway of CXCL1/CXCR2. Glial satellite cells induced by TNF-α were treated with simvastatin, 20 or 50 μM PCA, and there was no significant change in the phosphorylated JNK protein level in different treatment groups (p>0.05) (Figure 7A), while the expression of CXCL1, CXCR2, phosphorylated ERK and CREB, c-fox protein was upregulated in cells induced by TNF-α (p<0.01) (Figure 7B-E). In addition, PCA reversed the phosphorylation of ERK protein, CREB protein and the increase of CXCL1, CXCR2 and c-fox proteins expression induced by TNF-α (F_(4,10) p-ERK_=293.30; F_(4,10) CREB_=326.10; F_(4,10) CXCL1_=456.50; F_(4,10) CXCR2_=786.30; F_(4,10) c-fox_=590.20; p< 0.05) (Figure 7B-E). These results indicated that PCA could block the signal transduction of CXCL1/CXCR2/c-fox in the JNK pathway.

### PCA Inhibited the Expression of the JNK/CXCL1/CXCR2 Pathway Related Proteins in CCI Rat DRG

**FIGURE 8:**
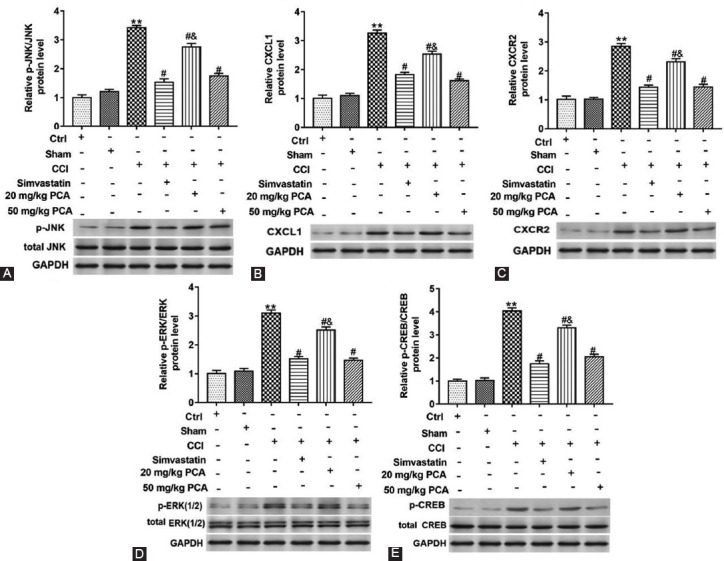
Effect of protocatechuic acid (PCA) on the JNK/CXCL1/CXCR2 pathway related protein expression in dorsal root ganglia (DRG) of chronic constriction injury (CCI) rats. Rats were treated with CCI, simvastatin or two doses (20 or 50 mg/kg) of PCA. Phosphorylation of ERK protein, CREB protein and c-fox protein levels in DRG of rats were detected with Western blot assay (A-E). **p<0.01 versus ctrl group or sham group, ^#^p<0.05 versus CCI group, CCI group treated with simvastatin or CCI group treated with 20 mg/kg PCA, ^&^p< 0.05 versus CCI group treated with simvastatin.

Finally, we validated the effect of PCA on the JNK/CXCL1/CXCR2 pathway related protein expression *in vivo*. Rats were treated with CCI alone or together with simvastatin, 20 or 50 mg/kg PCA, and we found that the expression of CXCL1, CXCR2, phosphorylated JNK and ERK, CREB protein, and c-fox proteins was upregulated in CCI rats (p<0.01) (Figure 8A-E). In addition, PCA reversed the phosphorylation of JNK protein and ERK protein, CREB protein and the increase of CXCL1, CXCR2, and c-fox proteins expression in CCI rats (F_(5,12)JNK_=285.60;F_(5,12)ERK_=234.20; F_(5,12)CREB_=362.60; F_(5,12)CXCL1_=265.60; F_(5,12)CXCR2_ =185.30; F_(5,12)c-fox_ =555.40; p<0.05) (Figure 8A-E). These results proved *in vivo* that PCA could reverse the activation of CCI on the JNK pathway, so as to alleviate the neural pain.

## DISCUSSION

In our study, we first constructed the CCI model of rats, and then we verified the relieving effect of PCA on the immune stress of CCI rats *in vivo* and *vitro*, and investigated that PCA inhibited the sciatic nerve pain by blocking the activation of JNK/CXCL1/CXCR2 signaling pathway. PCA is a natural phenolic antioxidant, which is used to prevent neurodegenerative diseases such as Alzheimer’s disease and Parkinson’s disease, and it has a beneficial effect on the accumulation of b-amyloid plaques in brain tissues [30]. In addition, PCA can improve the prognosis of mice with intracerebral hemorrhage by inhibiting oxidative stress, inflammation, and cell apoptosis [31]. Moreover, one study has shown that orally administration of PCA (30 mg/kg) for 3 consecutive days can alleviate the brain injury induced by epilepsy in adult male rats [14]. PCA at 30 and 50 mg/kg can significantly improve the behavior of adult male Wistar rats after focal cerebral ischemia caused by middle cerebral artery occlusion, and reduce infarction [25]. In this study, we found that PCA reversed the proliferation of glial satellite cells induced by TNF-α. Moreover, PCA alleviated pain in CCI rats in a concentration dependent manner, and 50 mg/kg PCA was better than 20 mg/kg PCA. A clinical study has shown that chronic pain is often related to the adaptation of brain networks such as emotion, motivation, and reward [32]. Therefore, we measured the thermal stimulation and mechanical foot contraction response of rats in different treatments *in vivo*. Our results revealed that CCI reduced TWL and PWMT, which was consistent with the results of other studies of chronic nerve injury [33]. Peripheral nerve injury leads to activation of corresponding DRG macrophages and local spinal cord inflammation through activation of glial cells, recruiting and releasing TNF-α, IL-6, or monocyte chemoattractant protein-1 by immune cells. Our study investigated the antioxidative indexes and cytokine levels of nerve tissue in CCI rats, and found that CCI rats reduced SOD content, but increased the contents of MDA, IL-1β, IL-6, and TNF-α. The results showed that IL-1β level was increased after peripheral nerve injury in rodents [34], which was consistent with our results. In addition, IL-1β level was also increased significantly in mice with memory impairment and depression after peripheral nerve injury [35]. Repair and regeneration after nerve injury involve a complex process of change, including inflammatory reaction, adhesion reaction, ECM synergy, regulation of neurotrophic factors on regeneration, synthesis and release of neurotransmitters, formation and extension of growth cones, and regeneration of neurons. It has been reported that drug treatment can reduce the aggregation of astrocytes and microglia in spinal dorsal horn and the infiltration of macrophages into dorsal root ganglion and sciatic nerve [36]. Anethole inhibited the activation of glial cells, decreased the secretion of pro-inflammatory cytokines to relieve the chronic nerve injury in mice [37]. PCA increased SOD content while decreased contents of MDA, IL-1β, IL-6, and TNF-α in CCI rats. At the cellular level, PCA has also been shown to reduce pain caused by CCI. Therefore, we can speculate that PCA and anethole have similar relieving effects on CCI induced neuropathic pain in rats. CXCL1 is mainly produced by endothelial cells and pericytes stimulated by TNF-α and supports the crawling of neutrophils in the lumen and subepithelial cells [38]. TNF-α upregulated the expression of CXCL1/2 in cancer cells through the NF-κB signaling pathway, thereby amplifying the CXCL1/2-S100A8/9 loop and causing chemoresistance [39]. In a mouse steatohepatitis model, it was found that the deletion of PPARγ gene highly enhanced the induction of palmitic acid or TNF-α on CXCL1 in mouse liver cells [40]. Kupffer cells in the liver can sense the damage-related molecular pattern molecules released by necrotic cells, and release TNF-α to activate the NF-κB pathway to upregulate CXCL1 level in hepatocytes [41]. In addition, it was detected in a rat model of neuropathic pain that Loganin prevents neuropathic pain caused by chronic contractile injury by reducing TNF-α/IL-1β-mediated NF-κB activation and Schwann cell demyelination [42]. Therefore, we can speculate that TNF-α upregulates CXCL1 level in CCI rats by activating the NF-κB signaling pathway. DRG contains the cell body of neurons that transmit sensory information from surrounding areas to the central nervous system through the dorsal and anterolateral tracts of the spinal cord. Each sensory neuron is surrounded by the cell bodies and layered processes of several satellite glial cells, forming a morphological and functional unit. Satellite glial cells play an important role in regulating the function of sensory neurons, especially in controlling the neuron microenvironment. Satellite cells are the main glial cells in DRG and also play an important role in the occurrence/regulation of chronic pain [43]. In addition, it has been reported that TNF-α can promote the activation of microglia and the development of CCI [44]. In this experiment, TNF-α was used to induce glial satellite cells to simulate the stimulation of sciatic nerve injury. *In vitro* experiment, the change trend of oxidative stress index and cytokine level in the supernatant of glial satellite cells was similar to that *in vivo*, and CXCL1 mRNA and protein level, its specific chemokine, and its receptor were increased in TNF-α induced glial satellite cells. Three pathways including the JNK, ERK, and p38/MAPK signaling pathways have been reported in the treatment of nerve injury [36,45-48]. In addition, the NF-κB signaling pathway is also reported as being involved in nerve injury. Our study further explores whether PCA can inhibit the NF-κB signaling pathway and investigates the mechanism of PCA in relieving CCI pain. We treated glial satellite cells with three kinds of pathway inhibitors respectively, and verified that PCA could alleviate the proliferation of glial satellite cells induced by TNF-α by blocking the JNK signaling pathway.

CXCL1 and its receptor CXCR2 have been reported in colon cancer and gastric cancer and play an important role in metastasis [47,49]. In addition, CXCL1 could be a diagnostic factor to observe whether high-grade prostate cancer has deteriorated [15], and the CXCL1/CXCR2 axis has been reported in the glial cells during neuropathic pain [21]. In paclitaxel induced neuropathic pain in mice, the expression levels of CXCL1 and its receptor CXCR2 were upregulated [20]. Moreover, downregulation of CXCL1 was used to relieve ischemic brain injury [50]. Similarly, our results showed that PCA reversed the upregulation of CXCL1 and CXCR2 protein levels in CCI rats. Consequently, we can speculate that PCA can block the JNK signal transmission to relieve nerve pain in CCI rats.

## CONCLUSION

PCA can improve CCI-induced neuroinflammation and pain behavior by inhibiting the JNK/CXCL1/CXCR2 signaling pathway.
